# Pulmonary Embolism and Right Ventricular Dysfunction: Mechanism and Management

**DOI:** 10.7759/cureus.70561

**Published:** 2024-09-30

**Authors:** Ogbonnaya N Ajah

**Affiliations:** 1 Department of Emergency Medicine, Barnet General Hospital, London, GBR

**Keywords:** anticoagulants, hemodynamics, heart ventricles, management, mechanisms, right ventricular dysfunction, pulmonary embolism

## Abstract

Pulmonary embolism (PE) occurs when thrombi from deep vein thrombosis dislodge and obstruct pulmonary arteries, raising pulmonary artery pressure and straining the right ventricle. This strain can lead to right ventricular dysfunction (RVD), characterized by reduced cardiac output, impaired contractility, and potential development of chronic thromboembolic pulmonary hypertension. Clinically, PE may present with symptoms such as dyspnea, pleuritic chest pain, and tachycardia. Diagnosis is typically confirmed through computed tomography pulmonary angiography, biomarkers like D-dimer and cardiac troponins, and clinical scoring systems. Acute management focuses on hemodynamic support, including intravenous fluids and vasopressors, and may involve anticoagulation with low-molecular-weight heparin or direct oral anticoagulants. Severe cases may require systemic anticoagulation, catheter-directed techniques, and surgeries like pulmonary endarterectomy. Long-term management involves continued anticoagulation tailored to individual risk factors, with ongoing monitoring to prevent recurrence. Effective early diagnosis and management are crucial, as severe PE can significantly increase mortality and lead to serious complications. This review explores the pathophysiology, diagnosis, and management of PE and RVD.

## Introduction and background

Pulmonary embolism (PE) is a severe and frequently life-threatening condition resulting from the obstruction of a pulmonary artery or its branches, typically due to a thrombus that has migrated from deep vein thrombosis (DVT) [1]. This obstruction disrupts blood flow to the lungs, causing an acute elevation in pulmonary arterial pressure. The resultant increase in pressure imposes substantial strain on the right ventricle (RV), the cardiac chamber responsible for propelling blood into the pulmonary circulation. As the RV attempts to manage the heightened workload, it may become overwhelmed, leading to right ventricular dysfunction (RVD). This dysfunction compromises the heart's ability to pump blood effectively, potentially resulting in reduced cardiac output, hypotension, and further systemic circulatory impairment. Significant hemodynamic compromise can occur in up to 8% of patients with acute PE. This condition is linked to a substantial rise in mortality rates, ranging from 15% to 42%, primarily due to acute right ventricular failure [2]. Clinically, PE can present with acute symptoms such as sudden onset of dyspnea, sharp chest pain, tachycardia, and in severe cases, syncope or collapse [3]. Long-term complications, including chronic thromboembolic pulmonary hypertension (CTEPH), can arise when elevated pulmonary pressures persist, leading to sustained RV impairment and ongoing cardiovascular strain [4].

The clinical significance of PE and RVD is substantial due to their impact on patient outcomes and healthcare systems. PE is a common disorder with an estimated yearly incidence of 0.7 to 1 per 1000 inhabitants in the Western world [5]. It is a leading cause of cardiovascular morbidity and mortality globally, with prevalence influenced by variables such as age, underlying health conditions, and lifestyle factors. RVD plays a critical role in the severity of PE and is associated with increased mortality and adverse outcomes. Patients exhibiting RVD face a higher risk of severe complications, including shock and death, and often require more intensive therapeutic interventions. In-hospital mortality due to PE ranges from 22.0% to 31.8%, increasing up to 65% in cases of cardiac arrest [6], with most deaths occurring within the first hour [7]. This highlights the critical need for early recognition and treatment. Research indicates that the presence of RVD is correlated with poorer prognosis and can complicate the management of PE, underscoring the importance of early detection and timely treatment [8]. A comprehensive understanding of the prevalence and impact of RVD in PE patients is essential for developing effective treatment strategies and enhancing patient outcomes. This review aims to comprehensively evaluate the mechanisms and management strategies associated with PE and RVD by analyzing the evidence from the current available literature. The objectives include examining the pathophysiological processes through which PE induces RVD, assessing the clinical impact of RVD on patient outcomes, and reviewing current treatment approaches.

## Review

Discussion

Pathophysiology and Mechanism

Pulmonary embolism: Most PEs arise from thrombi in the deep veins of the lower extremities, primarily in the calf veins, followed by the femoropopliteal veins, and less frequently in the iliac veins. Thrombosis often occurs in areas with reduced blood flow, such as near valve cusps and at bifurcations, and can be exacerbated by local hypercoagulability due to factors like hypoxia and hemoconcentration [9]. A smaller fraction of emboli originates from upper extremity veins, typically associated with central venous catheters, intracardiac devices such as pacemakers and defibrillators, malignancy, or trauma related to activity. Additionally, DVT in the pelvic veins can lead to PEs, especially when accompanied by risk factors such as pelvic infections, surgeries, or pregnancy. Central DVTs in the lower extremities are more likely to result in PE for 15%-32%, while those in the upper extremities have a lower incidence of PE approximately 6% [9].

PE occurs when clots formed in the deep venous system break loose and travel to block the pulmonary arteries. Smaller PEs may resolve on their own, but larger clots can cause a rapid and significant increase in pulmonary arterial pressure, potentially leading to circulatory collapse. Clot formation often begins at sites of tissue or vascular injury, where activated monocytes expose tissue factor, disrupting the body’s natural anticoagulant and fibrinolytic processes. DVT composed mainly of red blood cells, platelets, and fibrin typically forms in the venous sinuses or valve cusps of the lower extremities. The primary risk is that part or all of a DVT could travel to the lungs. Moreover, the congestion caused by DVT can lead to swelling and discomfort in the affected limb, along with impaired valve function. This dysfunction can obstruct blood flow, resulting in increased swelling, venous stasis, and further thrombus formation [10]. Risk factors include immobility, recent surgery, malignancy, pregnancy, and hormone use [11]. Virchow's triad endothelial injury, hypercoagulability, and venous stasis play a role in thrombus formation [12]. The obstruction increases pulmonary artery pressure, burdening the RV and potentially leading to RV dysfunction and reduced systemic perfusion [13]. Chronic obstruction can cause pulmonary hypertension and right ventricular dysfunction.

Right ventricular dysfunction: PE leads to increased pulmonary artery pressure and afterload on the RV, which struggles to generate adequate pressure [14]. Increased pulmonary vascular resistance (PVR) decreases RV stroke volume, causing acute dilation and impaired contractility, potentially leading to RV failure. Prolonged pressure overload can result in maladaptive RV remodeling, including dilation and reduced systolic function. Disrupted RV-arterial coupling affects contractility and relaxation, contributing to symptoms like peripheral edema and ascites [3]. The RV’s thin wall and low-pressure handling make it vulnerable to sudden increases in pressure. Acute PE leads to RV pressure overload through increased PVR and neurohormonal responses, even with low clot burdens [15]. Increased PVR causes decreased stroke volume and compensatory tachycardia, while fibrosis and inflammation further impair RV function [16]. RV dilation and interventricular septum displacement reduce left ventricle (LV) filling and systemic perfusion, exacerbating right-sided heart failure and creating a feedback loop of the RV and LV dysfunction.

Classification, Clinical Presentation and Diagnosis

The severity of PE is generally classified following the 2019 guidelines from the American Heart Association (AHA) and the European Society of Cardiology (ESC). These frameworks, which are quite similar, categorize PE into three main levels [17]. PE is classified into three categories based on severity and risk factors. Massive; high-risk PE, which accounts for about 5% of cases, is characterized by hypotension, defined as a systolic blood pressure below 90 mm Hg or a significant drop in blood pressure for at least 15 minutes, often requiring vasopressor support; this group has a mortality rate of approximately 65% [17-19]. Intermediate-risk; submassive PE accounts for about 40% of cases and includes patients showing signs of right ventricular strain, dilation, or dysfunction, with varying degrees of severity affecting mortality rates of 5-25% [17-19]. Low-risk PE, which encompasses 40-60% of cases, involves patients who do not meet the criteria for the other two categories, resulting in a much lower mortality rate of around 1%. This classification helps guide treatment and management strategies for patients with PE [17-19]. 

PE is often referred to as the great masquerade, as it can present with a wide range of symptoms [20]. Patients with symptomatic PE are at a greater risk for experiencing recurrent PE and fatal recurrent PE compared to those with DVT alone. Approximately one-third of individuals with acute DVT have asymptomatic PE. The prevalence of asymptomatic PE is roughly twice as high in patients with proximal DVT involving the popliteal vein or more proximal veins compared to those with distal DVT confined to the calf veins. Additionally, patients with unprovoked DVT are more likely to have asymptomatic PE than those with provoked DVT [21]. Commonly reported symptoms include dyspnea, pleuritic chest pain, cough, and hemoptysis, while tachypnea and tachycardia are frequently observed signs. Additionally, other manifestations such as syncope and alterations in mental status may occur [20]. Dyspnea ranges from mild to severe, while pleuritic chest pain is sharp and worsens with respiration. Tachycardia reflects the heart’s response to elevated pulmonary artery pressure and reduced cardiac output. Other signs include hypoxemia, jugular venous distention, and peripheral edema [22]. Atypical symptoms like syncope, hemoptysis, and nonspecific fatigue can complicate diagnosis. Chronic or recurrent PE may present as CTEPH with progressive dyspnea and right heart failure signs.

CT pulmonary angiography (CTPA) is the primary diagnostic tool, offering high sensitivity and specificity through detailed imaging of the pulmonary arteries [23]. Despite its accuracy, CTPA requires careful consideration of contrast use and radiation exposure. Echocardiography, including transesophageal echocardiography and transthoracic echocardiography, provides insights into right ventricular function and thrombi presence, though it is less definitive than CTPA. Biomarkers such as D-dimer, cardiac troponins, and B-type natriuretic peptide (BNP) aid in diagnosis. D-dimer is sensitive but lacks specificity for PE. Elevated cardiac troponins indicate significant RV strain and worse prognosis, while BNP reflects RV dysfunction and heart failure [24]. Recently, several prediction rules have been developed to categorize patients with suspected PE based on clinical or pretest probability. Among these, the Wells score and the revised Geneva score are the most validated and widely utilized, stratifying patients into low, moderate, and high probability groups. While both scoring systems have proven valuable for accurate diagnosis, there is limited research focusing on elderly patients. As per Guo et al., the Wells score demonstrates superior diagnostic accuracy compared to the revised Geneva score for those with suspected PE. Additionally, combining either score with a normal D-dimer level is an effective strategy for safely ruling out PE [25].

The management of patients with intermediate and high-risk PE is a complex and often debated issue. With various treatment options available, determining the most appropriate approach can be challenging. Additionally, care for PE patients frequently involves multiple medical specialties. To streamline the response to acute PE cases, many hospitals and academic institutions have established pulmonary embolism response teams (PERTs). These teams consist of a multidisciplinary group of experts in thromboembolic disease who can quickly address the needs of patients with acute PE. The objective of a PERT is to develop a rapid, individualized treatment plan without the need to consult several different specialists, drawing inspiration from the structure of rapid response teams [26].

Management

The acute management of PE involves a comprehensive approach to initial assessment and stabilization, with a particular focus on hemodynamic support. Upon presentation, immediate assessment of the patient’s hemodynamic status is critical. This includes evaluating vital signs such as blood pressure, heart rate, and oxygen saturation, as well as assessing for signs of shock, hypotension, or right ventricular strain [27]. The goal is to promptly identify and address any life-threatening conditions, such as severe hypotension or impaired cardiac output, which may require urgent intervention.

Hemodynamic support often involves the administration of intravenous fluids to maintain adequate blood volume and improve systemic perfusion. In cases where fluid resuscitation alone is insufficient to stabilize blood pressure, vasopressor agents may be required to augment cardiovascular function [28]. Commonly used vasopressors include norepinephrine, epinephrine, and dobutamine, which help restore adequate blood pressure and ensure sufficient perfusion of vital organs. The choice and dosage of these agents are tailored to the patient’s specific clinical condition and response to therapy. Additionally, a progressive rise in pulmonary arterial pressure caused by PE leads to ongoing deterioration in pulmonary artery hemodynamics, right ventricular function, right ventricular-pulmonary arterial coupling, and overall cardiac output. Levosimendan has been shown to more effectively restore right ventricular-pulmonary arterial coupling compared to placebo, primarily due to its dual effects of pulmonary vasodilation and enhanced right ventricular contractility [29]. Levosimendan is a calcium sensitizer that exhibits inotropic, pulmonary vasodilatory, and cardioprotective effects [30].

Historically, the management of high-risk PE has primarily centered on the prompt initiation of anticoagulation therapy, utilizing either unfractionated heparin (UFH) or low-molecular-weight heparin (LMWH). The main objective of this approach is to prevent the further propagation of blood clots and their migration to other areas of the body. While effective in certain cases, this strategy has led clinicians to explore alternative methods that more aggressively address the clot burden [31]. One such method is thrombolysis, which employs fibrinolytic agents to rapidly dissolve obstructive clots and restore pulmonary blood flow. However, the use of thrombolysis in MPE remains controversial. While it offers the potential for rapid clot resolution, there are significant concerns regarding the risk of bleeding, especially in hemodynamically unstable patients. This has led to ongoing debates within the medical community [31]. To evaluate the appropriateness of thrombolytic therapy, it is crucial to weigh the benefits of swift clot dissolution against the potential bleeding risks. This assessment should take into account individual patient characteristics, comorbidities, and the overall risk-benefit balance of the treatment. Conversely, reliance solely on anticoagulation presents limitations, as these medications primarily prevent further clot formation but may not sufficiently reduce the size of existing clots, potentially leading to ongoing circulation issues and associated complications [32,33].

Administered subcutaneously, LMWH typically requires daily or twice-daily injections, with dosage based on patient weight and renal function. This approach is favored for its predictable pharmacokinetics and reduced need for routine monitoring. UFH is another option, particularly for patients with severe PE or those requiring rapid reversal of anticoagulation [34]. Unfractionated heparin is given intravenously, starting with a bolus dose followed by continuous infusion, with dosing adjusted based on activated partial thromboplastin time or anti-factor Xa levels. While UFH requires frequent monitoring and dose adjustments, it allows for rapid titration and is suitable for high-risk patients or those undergoing invasive procedures. Direct oral anticoagulants (DOACs), such as rivaroxaban, apixaban, and edoxaban, are used for long-term management following initial therapy with LMWH or UFH [35]. These agents, administered orally, offer the advantage of fixed dosing and minimal need for routine monitoring. The transition from injectable anticoagulants to DOACs typically occurs after the initial stabilization period. Warfarin, a vitamin K antagonist, has traditionally been used for long-term anticoagulation but requires careful monitoring of the international normalized ratio (INR) and is often overlapped with LMWH or UFH until therapeutic levels are reached [36]. Despite its effectiveness, warfarin's use has declined in favor of DOACs due to their convenience and reduced monitoring requirements. Each anticoagulant has specific protocols and considerations, with treatment choices tailored to the patient’s condition and clinical circumstances.

Administration of thrombolytics typically involves intravenous infusion of agents such as tissue plasminogen activator (tPA), including alteplase, which is the most commonly used thrombolytic agent for PE [37]. The standard protocol involves a bolus dose followed by a continuous infusion over a specific duration, generally 1 to 2 hours, depending on the agent used and patient characteristics. Close monitoring during and after administration is crucial to detect potential complications, including bleeding and allergic reactions, and to assess the therapeutic response. Dosage adjustments and supportive measures, including blood pressure management and hemostatic monitoring, may be necessary to ensure patient safety and maximize treatment efficacy. However, the use of thrombolytic therapy is associated with several contraindications that must be carefully evaluated to minimize the risk of adverse effects [38]. Absolute contraindications include active bleeding or a recent history of bleeding disorders, intracranial hemorrhage, or recent major surgery, as these conditions significantly increase the risk of severe hemorrhagic complications. Other relative contraindications include a history of stroke or transient ischemic attack, uncontrolled hypertension, or significant trauma, which may warrant cautious consideration and thorough risk assessment before initiating therapy. However, the use of thrombolytic therapy is associated with several contraindications that must be carefully evaluated to minimize the risk of adverse effects [38]. Table 1 lists the absolute and relative contraindications for thrombolytic therapy, which may warrant cautious consideration and thorough risk assessment before initiating treatment [38,39].

**Table 1:** Absolute and Relative Contraindication to Thrombolytic Therapy

Absolute Contraindications	Relative Contraindications
Any previous history of hemorrhagic stroke	Systolic BP >180 mm Hg
History of stroke, dementia, or central nervous system damage within one year	Diastolic BP >110 mm Hg
Head trauma or brain surgery within six months	Recent non-intracranial bleeding
Known intracranial neoplasm	Recent surgery/invasive procedure
Suspected aortic dissection	Ischemic stroke >3 months
Active bleeding or known bleeding disorder	Anticoagulation
Major surgery, trauma, or bleeding within six weeks	Recent trauma from 2-4 weeks ago, including traumatic CPR
Ischemic stroke within three months	Pregnancy or within 1 week postpartum
Tendency to bleed	Uncompressible vascular bleeding
	Active peptic ulcer
	Acute pancreatitis
	Infective endocarditis
	Active cavitating pulmonary tuberculosis
	Advanced liver disease
	Intracardiac thrombi
	Puncture of noncompressible blood vessels within 2 weeks
	Previous streptokinase therapy
	Age >65
	Low body weight
	Female
	Black race

In the management of severe PE, surgical and catheter-based interventions may be necessary, particularly when thrombolytic therapy is contraindicated, ineffective, or not feasible [1]. These interventions aim to rapidly remove or dissolve thrombi to restore pulmonary blood flow and mitigate hemodynamic compromise. Embolectomy, a catheter-based procedure, involves the direct removal of emboli from the pulmonary arteries. It is typically considered in cases of massive PE or when there is a contraindication to thrombolytic therapy, such as a high risk of bleeding or when thrombolytics have failed to alleviate symptoms. The procedure can be performed via open surgery or minimally invasive techniques, such as catheter-based embolectomy. During open surgical embolectomy, a cardiopulmonary bypass is used to temporarily take over the function of the heart and lungs while the emboli are removed [40]. This approach is generally reserved for patients with severe hemodynamic instability or those who do not respond to other treatments. Minimally invasive techniques, such as the percutaneous approach, involve the use of specialized catheters and tools to remove thrombi through a small incision, often with a quicker recovery time and fewer complications compared to open surgery. Catheter-directed thrombolysis is a less invasive alternative to systemic thrombolysis and is used primarily for patients with intermediate-risk PE or those who are not candidates for systemic thrombolytics [1]. This technique involves the insertion of a catheter through a large vein, usually in the groin, which is then advanced to the pulmonary arteries. Thrombolytic agents, such as tPA, are delivered directly to the site of the clot via the catheter, allowing for targeted dissolution of the thrombus. The procedure may be performed using various methods, including mechanical thrombolysis devices that enhance the effectiveness of drug delivery or directly remove thrombi through mechanical means. The primary advantage of catheter-directed thrombolysis is its ability to reduce systemic bleeding risks compared to systemic thrombolysis while providing localized thrombus resolution [41].

Both embolectomy and catheter-directed thrombolysis require careful patient selection and consideration of risks versus benefits. These interventions are typically reserved for severe cases of PE where immediate restoration of pulmonary circulation is critical. Observational studies indicate that catheter-directed therapy may lower the risk of significant in-hospital bleeding compared to systemic thrombolysis, potentially leading to decreased in-hospital mortality and shorter stays in intensive care and overall hospital length. If ongoing prospective randomized trials confirm these positive outcomes, this could translate into cost savings that should be factored into future health economic evaluations. However, the direct costs associated with catheter-directed thrombolysis for advanced PE will need to be evaluated based on each country’s hospital reimbursement system. These costs should be considered alongside potential benefits, such as a reduction in early complications, earlier hospital discharge, improved return to work and productivity, and prevention of long-term issues [42].

Supportive Measures for RV Dysfunction

Supportive measures for RVD in the context of PE are essential for stabilizing patients and improving hemodynamic and respiratory function. These interventions aim to alleviate the strain on the RV and ensure adequate oxygenation and ventilation. Oxygen therapy is a fundamental supportive measure for patients with RV dysfunction, particularly those with compromised oxygenation due to impaired pulmonary circulation [43]. Mechanical ventilation provides positive pressure ventilation to support or replace spontaneous breathing, thereby enhancing gas exchange and reducing the workload on the RV. The choice between invasive ventilation (endotracheal intubation) and non-invasive ventilation (such as bilevel positive airway pressure or continuous positive airway pressure) depends on the patient’s clinical condition and the degree of respiratory distress. Invasive mechanical ventilation may be necessary for patients with severe hypoxemic respiratory failure, altered mental status, or inability to protect their airway [44]. Proper ventilation settings are crucial to avoid ventilator-induced lung injury and to minimize hemodynamic instability. Extracorporeal membrane oxygenation represents a more advanced supportive measure for patients with severe RV dysfunction, especially when conventional therapies are inadequate [45].

Long-term anticoagulation is a critical component in the chronic management of PE to prevent recurrent thromboembolic events and to manage the risk of further complications. The duration and monitoring of anticoagulant therapy are tailored to the individual patient’s clinical condition, the underlying risk factors, and the initial PE severity. The duration of anticoagulant therapy for patients with PE generally depends on whether the event was provoked or unprovoked. For patients with a provoked PE, such as one associated with a transient risk factor (e.g., recent surgery or prolonged immobilization), anticoagulation is typically continued for a period of 3 to 6 months. In cases of unprovoked PE, or when the initial event occurs in the absence of obvious risk factors, longer-term anticoagulation is often recommended [46]. This duration can extend from 6 to 12 months or even longer, depending on the patient's risk of recurrence and bleeding. For patients with recurrent PE, active cancer, or persistent risk factors, indefinite anticoagulation may be necessary. The decision to continue or discontinue therapy is based on a risk-benefit assessment, considering the potential for recurrent thromboembolism versus the risk of anticoagulant-related bleeding. Monitoring is essential to ensure the efficacy and safety of long-term anticoagulant therapy. For patients on warfarin, a vitamin K antagonist, regular monitoring of the international normalized ratio (INR) is required to maintain therapeutic levels and avoid complications such as bleeding or thrombosis [47]. The INR is typically checked every 1 to 4 weeks, depending on the stability of the patient's INR levels and any recent changes in medication or diet. Direct oral anticoagulants, including rivaroxaban, apixaban, and edoxaban, generally require less frequent monitoring, as these agents have predictable pharmacokinetics and dosing regimens. However, periodic assessment of renal function and adherence to therapy is recommended to ensure optimal dosing and effectiveness. For patients with renal impairment, dose adjustments and more frequent monitoring may be necessary.

Prognosis and Outcomes

The short-term prognosis following an acute pulmonary embolism and RVD is largely determined by the severity of the embolic event and the timeliness of therapeutic interventions. Patients with severe pulmonary artery obstruction and significant RV dysfunction are at heightened risk for acute morbidity and mortality. Rapid and effective management is crucial to improving immediate survival rates and mitigating adverse outcomes during the acute phase of the condition [48]. Long-term prognosis for individuals with a history of pulmonary embolism and RVD is influenced by several factors, including the resolution of the acute event, the presence of residual pulmonary hypertension, and the efficacy of ongoing management strategies. While many patients achieve substantial recovery, some may endure persistent symptoms, diminished exercise capacity, and a reduced quality of life [49]. A study by Farmakis et al. concluded that three months following PE, 37% of patients experienced dyspnea, while 22% exhibited abnormal six-minute walk distance (6MWD). After one year, 20% continued to show abnormal 6MWD. There was a correlation between dyspnea and abnormal 6MWD, yet over half of those with abnormal 6MWD did not report experiencing dyspnea. Abnormal 6MWD was a significant predictor of later impairment and a decline in long-term quality of life after PE [50].

Additionally, studies with varying methodologies have shown that residual perfusion defects can be identified in up to 50% of patients six months after a PE through ventilation-perfusion lung scans. Approximately half of these patients continue to experience persistent symptoms and cardiopulmonary functional limitations for up to one-year post-PE. While there has been a growing focus on the long-term hemodynamic and functional effects of acute PE, the impact on patients' psychological and emotional well-being, as well as their quality of life, remains under-researched. Health crises like an acute PE can lead to emotional distress, which may manifest as anxiety, anger, depression, or post-traumatic stress disorder. In individuals with chronic heart failure and pulmonary artery hypertension, a diminished quality of life has been linked to poorer prognosis and has become a key focus in treatment strategies for chronic heart failure [51].

The long-term survival impact is significant, particularly for those who develop CTEPH or experience complications such as chronic right heart failure. Predictors of adverse outcomes include residual pulmonary hypertension, severe RV dysfunction, and comorbid conditions such as heart failure or chronic obstructive pulmonary disease [52]. Advanced age, persistent symptoms despite treatment, and non-adherence to therapeutic regimens further exacerbate the risk of poor prognosis and recurrence. Identifying these risk factors is crucial for tailoring individualized treatment plans, implementing preventive measures, and improving long-term patient outcomes. Key findings and management of PE are illustrated in detail in Table 2.

**Table 2:** Comparative Analysis of Mechanism and Management Strategies in Acute Pulmonary Embolism and Right Ventricular Dysfunction PE: Pulmonary Embolism; RV: Right Ventricular; ECMO: Extracorporeal Membrane Oxygenation; NO: Nitric Oxide; V/Q: Ventilation/Perfusion *The Pulmonary Embolism Severity Index (PESI) and the BOVA are scoring systems used to assess the severity and prognosis of patients with PE. PESI categorizes patients based on clinical factors providing a score that correlates with 30-day mortality risk. Both scores aim to stratify patients effectively, aiding clinicians in determining appropriate management strategies for PE [65].

Author	Mechanism and Key Findings	Management
Schouver et al. [53]	Diuretics improved RV function markers faster by reducing fluid overload and RV strain in patients with submassive PE and RV dysfunction; no severe outcome difference	Diuretics (furosemide) vs. volume expansion (saline)
Ternacle et al. [54]	Diuretics reduced shock index and blood pressure in acute RV failure patients, while fluid expansion had no significant hemodynamic effects	Diuretics (furosemide) vs. isotonic saline
Gallet et al. [55]	Identified predictors of RV failure and high mortality with elevated pulmonary artery pressure; managed with diuretics and vasodilators	Diuretics, vasodilators, supportive care
Boulain et al. [56]	Epinephrine improved hemodynamics and RV ejection fraction in a woman with shock and acute PE; prior treatments were ineffective	IV epinephrine infusion
Jardin et al. [57]	Dobutamine improved cardiac index and reduced pulmonary vascular resistance in patients with massive PE and circulatory failure	IV dobutamine infusion, blood volume expansion
McIntyre et al. [14]	Hypoxemia and elevated pressures reflect RV strain, influencing management strategies in male patients with confirmed PE	Hemodynamic monitoring; implications for thrombolytic therapy
Alpert et al. [16]	Minor PE caused higher pulmonary pressures and lower PaO₂ due to hypoxemia and vasoconstriction	Oxygen therapy; further research needed
Hirsh et al. [58]	Streptokinase improved angiography and hemodynamics more effectively than heparin in severe PE patients	Streptokinase vs. heparin
Kooter et al. [59]	Epoprostenol did not significantly affect RV diameter in patients with acute PE and RV dilatation, showing limited effectiveness for reducing pulmonary pressure	Conventional treatment with or without epoprostenol
Szold et al. [60]	Inhaled NO improved pulmonary and systemic blood pressures and gas exchange in patients with massive PE	Inhaled NO with conventional treatments
Kline et al. [61]	NO showed limited efficacy in improving RV function compared to placebo in patients with acute PE and RV dysfunction	Anticoagulation with NO or placebo
Huet et al. [22]	V/Q mismatch and intrapulmonary shunt caused hypoxemia in severe PE patients, affecting respiratory function	Diagnostic findings; no specific management strategies
Yusuff et al. [62]	ECMO had a 70.1% survival rate; higher death risk with ECMO during cardiac arrest in severe PE cases	ECMO, thrombolysis, surgical or catheter embolectomy
Shokr et al. [1]	Improved RV function and hemodynamics with Impella RP and thrombolysis in RV failure due to PE	Catheter-directed thrombolysis and Impella RP
Mavromanoli et al. [63]	Timely anticoagulation significantly improved RV function in 70% of intermediate-risk PE patients, reducing RV strain	Standard anticoagulation, supportive care
Chen et al. [64]	RV dysfunction alone is a sufficient marker for risk stratification in acute PE; PESI and BOVA scores may underestimate mortality*	Focus on RV dysfunction for treatment decisions

## Conclusions

Recent advancements in the management of PE and RVD are reshaping clinical practice. Prompt diagnosis, effective anticoagulation, and timely interventions are essential for enhancing patient outcomes. Innovations such as targeted anticoagulants, advanced interventional techniques, and personalized medicine offer the potential for more precise treatment strategies. To fully leverage these advancements, it is important to integrate novel therapies with individualized management approaches. Future research should prioritize evaluating the long-term efficacy of new treatments, refining personalized care, and optimizing management protocols to further improve patient outcomes.

## References

[1] Shokr M, Rashed A, Mostafa A, Mohamad T, Schreiber T, Elder M, Kaki A (2018). Impella RP support and catheter-directed thrombolysis to treat right ventricular failure caused by pulmonary embolism in 2 patients. Tex Heart Inst J.

[2] Jaff MR, McMurtry MS, Archer SL (2011). Management of massive and submassive pulmonary embolism, iliofemoral deep vein thrombosis, and chronic thromboembolic pulmonary hypertension: a scientific statement from the American Heart Association. Circulation.

[3] Riedel M (2001). Acute pulmonary embolism 1: pathophysiology, clinical presentation, and diagnosis. Heart.

[4] Simonneau G, Torbicki A, Dorfmüller P, Kim N (2017). The pathophysiology of chronic thromboembolic pulmonary hypertension. Eur Respir Rev.

[5] Yandrapalli S, Tariq S, Kumar J, Aronow WS, Malekan R, Frishman WH, Lanier GM (2018). Chronic thromboembolic pulmonary hypertension: epidemiology, diagnosis, and management. Cardiol Rev.

[6] Chopard R, Behr J, Vidoni C, Ecarnot F, Meneveau N (2022). An update on the management of acute high-risk pulmonary embolism. J Clin Med.

[7] Martinez Licha CR, McCurdy CM, Maldonado SM, Lee LS (2020). Current management of acute pulmonary embolism. Ann Thorac Cardiovasc Surg.

[8] Watts JA, Marchick MR, Kline JA (2010). Right ventricular heart failure from pulmonary embolism: key distinctions from chronic pulmonary hypertension. J Card Fail.

[9] Turetz M, Sideris AT, Friedman OA, Triphathi N, Horowitz JM (2018). Epidemiology, pathophysiology, and natural history of pulmonary embolism. Semin Intervent Radiol.

[10] Corrigan D, Prucnal C, Kabrhel C (2016). Pulmonary embolism: the diagnosis, risk-stratification, treatment and disposition of emergency department patients. Clin Exp Emerg Med.

[11] Dalen JE (2003). Economy class syndrome: too much flying or too much sitting?. Arch Intern Med.

[12] Jerjes-Sánchez C, Jerjes-Sánchez C (2015). Mechanisms of thrombosis. Thrombolysis in Pulmonary Embolism.

[13] Tapson VF (2008). Acute pulmonary embolism. N Engl J Med.

[14] McIntyre KM, Sasahara AA (1971). The hemodynamic response to pulmonary embolism in patients without prior cardiopulmonary disease. Am J Cardiol.

[15] Malik AB (1983). Pulmonary microembolism. Physiol Rev.

[16] Alpert JS, Godtfredsen J, Ockene IS, Anas J, Dalen JE (1978). Pulmonary hypertension secondary to minor pulmonary embolism. Chest.

[17] Russell C, Keshavamurthy S, Saha S (2022). Classification and stratification of pulmonary embolisms. Int J Angiol.

[18] Giri J, Sista AK, Weinberg I (2019). Interventional therapies for acute pulmonary embolism: current status and principles for the development of novel evidence: a scientific statement from the American Heart Association. Circulation.

[19] Konstantinides SV, Meyer G, Becattini C (2019). 2019 ESC Guidelines for the diagnosis and management of acute pulmonary embolism developed in collaboration with the European Respiratory Society (ERS): The Task Force for the diagnosis and management of acute pulmonary embolism of the European Society of Cardiology (ESC). Eur Respir J.

[20] Khasin M, Gur I, Evgrafov EV, Toledano K, Zalts R (2023). Clinical presentations of acute pulmonary embolism: a retrospective cohort study. Medicine (Baltimore).

[21] Boc A, Vene N, Košmelj K, Mavri A (2017). Impact of asymptomatic pulmonary embolism on the long-term prognosis of patients with deep venous thrombosis. Semin Thromb Hemost.

[22] Huet Y, Lemaire F, Brun-Buisson C, Knaus WA, Teisseire B, Payen D, Mathieu D (1985). Hypoxemia in acute pulmonary embolism. Chest.

[23] Prisco D, Grifoni E (2009). The role of D-dimer testing in patients with suspected venous thromboembolism. Semin Thromb Hemost.

[24] Chagnon I, Bounameaux H, Aujesky D (2002). Comparison of two clinical prediction rules and implicit assessment among patients with suspected pulmonary embolism. The. Am J Med.

[25] Guo DJ, Zhao C, Zou YD, Huang XH, Hu JM, Guo L (2015). Values of the Wells and revised Geneva scores combined with D-dimer in diagnosing elderly pulmonary embolism patients. Chin Med J (Engl).

[26] Channick RN (2021). The pulmonary embolism response team: why and how?. Semin Respir Crit Care Med.

[27] Pérez-Nieto OR, Gómez-Oropeza I, Quintero-Leyra A (2023). Hemodynamic and respiratory support in pulmonary embolism: a narrative review. Front Med (Lausanne).

[28] Rich S, Gubin S, Hart K (1990). The effects of phenylephrine on right ventricular performance in patients with pulmonary hypertension. Chest.

[29] Kerbaul F, Gariboldi V, Giorgi R (2007). Effects of levosimendan on acute pulmonary embolism-induced right ventricular failure. Crit Care Med.

[30] Hansen MS, Andersen A, Nielsen-Kudsk JE (2018). Levosimendan in pulmonary hypertension and right heart failure. Pulm Circ.

[31] Yamada N, Fukuda I, Nakamura M (2023). Prognostication of patients with pulmonary thromboembolism with and without residual deep vein thrombosis: a subanalysis of the J'xactly study. Ann Vasc Dis.

[32] Meyer G (2014). Effective diagnosis and treatment of pulmonary embolism: improving patient outcomes. Arch Cardiovasc Dis.

[33] Mohamad T, Kanaan E, Ogieuhi IJ (2024). Thrombolysis vs anticoagulation: unveiling the trade-offs in massive pulmonary embolism. Cureus.

[34] Malloy RJ, Rimsans J, Rhoten M, Sylvester K, Fanikos J (2018). Unfractionated heparin and low-molecular-weight heparin. Anticoagulation Therapy.

[35] Comerota AJ, Ramacciotti E (2016). A comprehensive overview of direct oral anticoagulants for the management of venous thromboembolism. Am J Med Sci.

[36] Shapiro NL, Bhatt SH (2016). Critical review and update on the treatment of acute and chronic pulmonary embolism. J Pharm Pract.

[37] Islam MS (2017). Thrombolytic therapy by tissue plasminogen activator for pulmonary embolism. Adv Exp Med Biol.

[38] Tapson VF (2013). Thrombolytic therapy for acute pulmonary embolism. Semin Thromb Hemost.

[39] Makki N, Brennan TM, Girotra S (2015). Acute coronary syndrome. J Intensive Care Med.

[40] Cross FS, Jones RD, Mowlem A (1964). Acute pulmonary embolism: surgical management using cardiopulmonary by-pass. Arch Surg.

[41] Bhatt A, Al-Hakim R, Benenati JF (2017). Techniques and devices for catheter-directed therapy in pulmonary embolism. Tech Vasc Interv Radiol.

[42] Mohr K, Keeling B, Kaier K (2024). Modelling costs of interventional pulmonary embolism treatment: implications of US trends for a European healthcare system. Eur Heart J Acute Cardiovasc Care.

[43] Friedman O, Horowitz JM, Ramzy D (2017). Advanced cardiopulmonary support for pulmonary embolism. Tech Vasc Interv Radiol.

[44] Yamamoto T (2018). Management of patients with high-risk pulmonary embolism: a narrative review. J Intensive Care.

[45] Baldetti L, Beneduce A, Cianfanelli L (2021). Use of extracorporeal membrane oxygenation in high-risk acute pulmonary embolism: a systematic review and meta-analysis. Artif Organs.

[46] Rodger M, Carrier M, Gandara E, Le Gal G (2010). Unprovoked venous thromboembolism: short term or indefinite anticoagulation? Balancing long-term risk and benefit. Blood Rev.

[47] Lee A, Crowther M (2011). Practical issues with vitamin K antagonists: elevated INRs, low time-in-therapeutic range, and warfarin failure. J Thromb Thrombolysis.

[48] Matthews JC, McLaughlin V (2008). Acute right ventricular failure in the setting of acute pulmonary embolism or chronic pulmonary hypertension: a detailed review of the pathophysiology, diagnosis, and management. Curr Cardiol Rev.

[49] Bejjani A, Khairani CD, Piazza G (2023). Right ventricular recovery: early and late changes after acute PE diagnosis. Semin Thromb Hemost.

[50] Farmakis IT, Valerio L, Barco S (2024). Functional capacity and dyspnea during follow-up after acute pulmonary embolism. J Thromb Haemost.

[51] Keller K, Tesche C, Gerhold-Ay A (2019). Quality of life and functional limitations after pulmonary embolism and its prognostic relevance. J Thromb Haemost.

[52] Weekes AJ, Johnson AK, Troha D, Thacker G, Chanler-Berat J, Runyon M (2017). Prognostic value of right ventricular dysfunction markers for serious adverse events in acute normotensive pulmonary embolism. J Emerg Med.

[53] Schouver ED, Chiche O, Bouvier P, Doyen D, Cerboni P, Moceri P, Ferrari E (2017). Diuretics versus volume expansion in acute submassive pulmonary embolism. Arch Cardiovasc Dis.

[54] Ternacle J, Gallet R, Mekontso-Dessap A (2013). Diuretics in normotensive patients with acute pulmonary embolism and right ventricular dilatation. Circ J.

[55] Gallet R, Meyer G, Ternacle J (2015). Diuretic versus placebo in normotensive acute pulmonary embolism with right ventricular enlargement and injury: a double-blind randomised placebo controlled study. Protocol of the DiPER study. BMJ Open.

[56] Boulain T, Lanotte R, Legras A, Perrotin D (1993). Efficacy of epinephrine therapy in shock complicating pulmonary embolism. Chest.

[57] Jardin F, Genevray B, Brun-Ney D, Margairaz A (1985). Dobutamine: a hemodynamic evaluation in pulmonary embolism shock. Crit Care Med.

[58] Hirsh J, McDonald IG, Hale GA, O'Sullivan EF, Jelinek VM (1971). Comparison of the effects of streptokinase and heparin on the early rate of resolution of major pulmonary embolism. Can Med Assoc J.

[59] Kooter AJ, Ijzerman RG, Kamp O, Boonstra AB, Smulders YM (2010). No effect of epoprostenol on right ventricular diameter in patients with acute pulmonary embolism: a randomized controlled trial. BMC Pulm Med.

[60] Szold O, Khoury W, Biderman P, Klausner JM, Halpern P, Weinbroum AA (2006). Inhaled nitric oxide improves pulmonary functions following massive pulmonary embolism: a report of four patients and review of the literature. Lung.

[61] Kline JA, Puskarich MA, Jones AE (2019). Inhaled nitric oxide to treat intermediate risk pulmonary embolism: a multicenter randomized controlled trial. Nitric Oxide.

[62] Yusuff HO, Zochios V, Vuylsteke A (2015). Extracorporeal membrane oxygenation in acute massive pulmonary embolism: a systematic review. Perfusion.

[63] Mavromanoli AC, Barco S, Ageno W (2023). Recovery of right ventricular function after intermediate-risk pulmonary embolism: results from the multicentre Pulmonary Embolism International Trial (PEITHO)-2. Clin Res Cardiol.

[64] Chen YL, Wright C, Pietropaoli AP (2020). Right ventricular dysfunction is superior and sufficient for risk stratification by a pulmonary embolism response team. J Thromb Thrombolysis.

[65] Murguia AR, Segovia F, Ayvali F (2024). Evaluation of four validated risk scores to predict outcomes in Hispanic patients with acute pulmonary embolism. Angiology.

